# Draft Genome Sequences of 12 *Mycolicibacterium smegmatis* Strains Resistant to Imidazo[1,2-*b*][1,2,4,5]Tetrazines

**DOI:** 10.1128/MRA.00263-19

**Published:** 2019-04-18

**Authors:** Aleksey A. Vatlin, Kirill V. Shur, Valery N. Danilenko, Dmitry A. Maslov

**Affiliations:** a Vavilov Institute of General Genetics, Russian Academy of Sciences, Moscow, Russia; Georgia Institute of Technology

## Abstract

Here, we report 12 draft genome sequences of mutant Mycolicibacterium smegmatis strains resistant to imidazo[1,2-*b*][1,2,4,5]tetrazines, which are antituberculosis drug candidates. We have identified 7 different mutations in the MSMEG_1380 gene, which encodes the AcrR/TetR_N transcriptional repressor, which may activate efflux-mediated resistance.

## ANNOUNCEMENT

We have previously described a number of substituted imidazo[1,2-*b*][1,2,4,5]tetrazines that showed promising MICs against both drug-susceptible and drug-resistant Mycobacterium tuberculosis strains. We used Mycolicibacterium smegmatis (formerly Mycobacterium smegmatis) as the model organism to study drug resistance mechanisms of mycobacteria to these compounds. We were able to obtain M. smegmatis mc2 155 spontaneous mutants that were resistant to 4 different imidazo[1,2-*b*][1,2,4,5]tetrazines at 3.5 to 4× MIC (1; D. A. Maslov, A. V. Korotina, K. V. Shur, A. A. Vatlin, O. B. Bekker, S. G. Tolshchina, R. I. Ishmetova, N. K. Ignatenko, G. L. Rusinov, V. N. Charushin, and V. N. Danilenko, unpublished data). We conducted whole-genome sequencing of the mutant strains and of wild-type M. smegmatis mc2 155 cultured in our laboratory. Here, we report the *de novo* assembly of a total of 13 M. smegmatis genomes, namely, 12 imidazo[1,2-*b*][1,2,4,5]tetrazine-resistant mutants and the original wild-type strain.

M. smegmatis strains were cultured in Middlebrook 7H9 medium with addition of oleic acid-albumin-dextrose-catalase (OADC; HiMedia, India) at 37°C for 16 to 40 h. Genomic DNA was isolated and purified by phenol-chloroform/isoamyl alcohol extraction after enzymatic cell lysis, as described in Belisle et al. (2). An NEBNext Ultra II DNA library prep kit for Illumina (New England Biolabs, USA) was used to prepare paired-end libraries. Quality control of the libraries was performed using gel electrophoresis and a Bioanalyzer 2100 instrument. The raw sequencing data were obtained using a HiSeq 2500 instrument (Illumina, USA) in rapid run mode with a HiSeq Rapid Sequencing by Synthesis (SBS) kit v. 2 (2 × 100 bp; Illumina, USA).

The read quality was checked with FastQC (v. 0.11.7) (3), which showed deviations in G+C content in the first 7 to 9 bases in most of the read archives and excessive adapter content in some of them, while Trimmomatic (v. 0.36) (4) was used for quality and adapter trimming (with the options HEADCROP:9 MINLEN:36 ILLUMINACLIP:TruSeq3-PE-2.fa:2:30:10 ILLUMINACLIP:TruSeq2-PE.fa:2:30:10 ILLUMINACLIP:NexteraPE-PE.fa:2:30:10 where applicable according to FastQC results). The reads were assembled to initial draft genomes by SPAdes (v. 3.13) with default settings (5). QUAST (v. 5.0.2) was used to assess the assembly metrics (6). The NCBI Prokaryotic Genome Annotation Pipeline was used for annotation. The resulting draft genome sequence metrics are shown in Table 1.

**TABLE 1:** Sequencing data for 13 M. smegmatis genomes

Organism	WGS (GenBank) accession no.	SRA accession no.	Genome size (bp)	Observed/predicted G+C content (mol%)	No. of proteins	Coverage (×)	No. of contigs >500 bp	*N*_50_ (kbp)
M. smegmatis mc2 155	SIJM00000000	SRR8589635	6,827,731	67.42/67.40	6,629	64.48	108	141,003
M. smegmatis atR1	SITP00000000	SRR8594823	6,835,158	67.43/67.40	6,641	72.00	99	143,573
M. smegmatis atR2	SITQ00000000	SRR8594824	6,829,205	67.43/67.40	6,620	65.24	99	170,775
M. smegmatis atR8	SITR00000000	SRR8594821	6,833,447	67.43/67.40	6,629	44.84	98	174,679
M. smegmatis atR9	SITS00000000	SRR8594822	6,831,345	67.43/67.40	6,625	58.88	99	170,384
M. smegmatis atR10	SITT00000000	SRR8594819	6,832,606	67.43/67.40	6,642	52.35	100	170,775
M. smegmatis atR11	SITU00000000	SRR8594820	6,830,272	67.43/67.40	6,637	81.68	107	140,853
M. smegmatis atR14	SITV00000000	SRR8594817	6,915,101	67.43/67.40	6,962	142.10	109	140,599
M. smegmatis atR17	SITW00000000	SRR8594818	6,830,791	67.43/67.40	6,627	88.34	96	143,574
M. smegmatis atR19	SITX00000000	SRR8594825	6,848,781	67.43/67.40	6,692	113.22	100	151,235
M. smegmatis atR33	SITY00000000	SRR8594826	6,835,795	67.43/67.40	6,651	42.60	122	116,922
M. smegmatis atR37	SITZ00000000	SRR8594827	6,832,326	67.43/67.40	6,631	67.51	99	143,573
M. smegmatis atR40	SIUA00000000	SRR8594828	6,827,014	67.43/67.40	6,647	66.98	133	101,803

As a result of a comparative bioinformatics analysis of M. smegmatis atR strain genomes, we were able to identify 7 different mutations in 11 strains (4 insertions, 2 deletions, and 1 single nucleotide substitution) in the MSMEG_1380 gene encoding the AcrR/TetR_N transcriptional regulator, which is involved in the regulation of gene expression of MmpS5/MmpL5 transmembrane transporters. It has been previously reported that overexpression of the *mmpS5*-*mmpL5* operon modulates resistance to the derivatives of thiacetazone in Mycobacterium abscessus (7) and cross-resistance to bedaquiline and clofazimine in M. tuberculosis (8).

### Data availability.

The whole-genome shotgun (WGS) assemblies have been deposited in NCBI GenBank; the versions described in this paper are the first versions. The read archives have been deposited in NCBI SRA. The WGS (GenBank) and SRA accession numbers are listed in Table 1. All of the data are part of BioProject identifier (ID) PRJNA454808, except those for M. smegmatis mc2 155 (BioProject ID PRJNA523130).

## References

[1] Maslov DA, Shur KV, Vatlin AA, Bekker OB, Korotina AV, Rusinov GL, Charushin VN, Danilenko VN 2018 Search for azolo[1,2,4,5]tetrazines biotargets in mycobacteria. FEBS OpenBio 8:263.

[2] Belisle JT, Mahaffey SB, Hill PJ 2010 Isolation of *Mycobacterium* species genomic DNA, p 1–12. *In* Parish T, Brown AC (ed), Mycobacteria protocols. Humana Press, Totowa, NJ. 10.1007/978-1-59745-207-6_1 20560080

[3] Andrews S 2010 FastQC: a quality control tool for high throughput sequence data. http://www.bioinformatics.babraham.ac.uk/projects/fastqc.

[4] Bolger AM, Lohse M, Usadel B 2014 Trimmomatic: a flexible trimmer for Illumina sequence data. Bioinformatics 30:2114–2120. doi:10.1093/bioinformatics/btu170. 24695404 PMC4103590

[5] Bankevich A, Nurk S, Antipov D, Gurevich AA, Dvorkin M, Kulikov AS, Lesin VM, Nikolenko SI, Pham S, Prjibelski AD, Pyshkin AV, Sirotkin AV, Vyahhi N, Tesler G, Alekseyev MA, Pevzner PA 2012 SPAdes: a new genome assembly algorithm and its applications to single-cell sequencing. J Comput Biol 19:455–477. doi:10.1089/cmb.2012.0021. 22506599 PMC3342519

[6] Gurevich A, Saveliev V, Vyahhi N, Tesler G 2013 QUAST: quality assessment tool for genome assemblies. Bioinformatics 29:1072–1075. doi:10.1093/bioinformatics/btt086. 23422339 PMC3624806

[7] Richard M, Gutiérrez AV, Viljoen AJ, Ghigo E, Blaise M, Kremer L 2018 Mechanistic and structural insights into the unique TetR-dependent regulation of a drug efflux pump in *Mycobacterium abscessus*. Front Microbiol 9:649. doi:10.3389/fmicb.2018.00649. 29675007 PMC5895659

[8] Hartkoorn RC, Uplekar S, Cole ST 2014 Cross-resistance between clofazimine and bedaquiline through upregulation of MmpL5 in *Mycobacterium tuberculosis*. Antimicrob Agents Chemother 58:2979–2981. doi:10.1128/AAC.00037-14. 24590481 PMC3993252

